# Transferrin-binding domain inserted-adenovirus hexon engineering enables systemic immune evasion and intratumoral T-cell activation

**DOI:** 10.7150/thno.105163

**Published:** 2025-01-01

**Authors:** Dae Hoon Lee, Youngtae Kwon, Ki Hwan Um, Jung Ki Yoo, Wootae Ha, Ki-Su Kim, Jintak Cha, Ha-Eun Cho, Kyung Sun Park, Min Jeong Kye, Jin Woo Choi

**Affiliations:** 1 Department of Pharmacology, College of Pharmacy, Kyung Hee University, Seoul 02447, Republic of Korea.; 2 Department of Biomedical and Pharmaceutical Sciences, College of Pharmacy, Kyung Hee University, Seoul 02447, Republic of Korea.; 3 R&D Center of Curigin Ltd. Seoul 04778, Republic of Korea.; 4 Department of Regulatory Science, College of Pharmacy, Kyung Hee University, Seoul 02447, Republic of Korea.; 5 Department of Laboratory Medicine, Kyung Hee University College of Medicine, Kyung Hee University Medical Center, Seoul 02447, Republic of Korea.

**Keywords:** systemic injectable viral vector, antibody evading viral vector, hexon engineered adenovirus, adenovirus, oncolytic virus

## Abstract

**Rationale:** Adenovirus-based therapies have encountered significant challenges due to host immunity, particularly from pre-existing antibodies. Many trials have struggled to evade antibody response; however, the efficiency of these efforts was limited by the diversity of antibody Fv-region recognizing multiple amino acid sequences.

**Methods:** In this study, we developed an antibody-evading adenovirus vector by encoding a plasma-rich protein transferrin-binding domain. The coding sequence was employed from *Neisseria Meningitides* and inserted in the experimentally-optimized site within the adenovirus capsid protein.

**Result:** This engineered antibody-evading oncolytic adenovirus overcame the reduction in productivity and infectivity typically caused by the insertion of a foreign domain. We observed decreased immune recognition and compromised formation of anti-adenovirus antibodies. Furthermore, the anti-tumor efficacy was demonstrated both *in vitro* and *in vivo*, with increased recruitment of CD8^+^ T cells.

**Conclusion:** This novel antibody-evading strategy effectively evades neutralizing antibodies and innate immunity while boosting cytotoxic immunity by recruiting CD8^+^ T cells at the tumor site. Additionally, this strategy holds potential for application in other gene therapies and adenovirus vectors.

## Introduction

Recently, diverse therapies utilizing viral vectors have emerged, primarily focusing on gene therapy and anti-cancer treatments. In the research field of cancer treatment using viral vectors, significant progress has been made since the US Food and Drug Administration (FDA) approved the first cancer therapeutic virus, talimogene laherparepvec (also known as T-VEC or Imlygic), for the local treatment of recurrent melanoma 1. Notable subsequent approvals include adenoviral vectors nadofaragene firadenovec-vncg (also known as Adstiladrin) 2 and cretostimogene grenadenorepvec 3 for non-muscle invasive bladder cancer that no longer responds to standard therapy. Following these approvals, a diverse array of research efforts continues to advance anti-cancer viral therapy 4-6. However, the application of these FDA-approved oncolytic viruses remains restricted to regions that can be directly injected, such as the skin via intratumoral injection or bladder via intravesical instillation. This administration strategy intends to avoid immune responses such as a cytokine release syndrome and an attack from neutralizing antibodies. In the field of gene therapy using viral vectors, the FDA has approved onasemnogene abeparvovec (Zolgensma) for the treatment of spinal muscular atrophy 7. Similar to previous virotherapies, it may face challenges due to neutralizing antibodies in circulation from its second injection, which can diminish the efficacy of the treatment 8. Consequently, most viral vector-based therapies still have a potential immune limitation and risk, depending on the specific condition of the patients.

Adenoviruses are known for their relatively low pathogenicity compared to other types of viruses in the field of oncolytic virotherapy 9. They typically cause only mild infectious symptoms that are self-limiting 10. In addition, in adverse effects, anti-adenoviral treatment with cidofovir can expect a rapid suppression of viral propagation 11. Thus, adenovirus may be a safer option for therapeutic use. Despite the safety profile of oncolytic adenoviruses, pre-existing neutralizing antibodies to adenovirus are a significant challenge to compromise the therapeutic efficacy. These antibodies are commonly found across most people over the continents and are present in high concentrations in mouse serum 12-14. While the high prevalence of antibodies suggests safety 15, 16, it still poses a hurdle for the effectiveness of oncolytic adenoviruses.

Numerous efforts to develop adenoviral vectors that can escape the immune system have focused on evading antibody recognition 17-19. However, these approaches, which typically involve modifying only a few amino acids, may offer limited efficacy in evading an attack of antibody as anti-adenoviral antibodies target multiple position poly clonally, not just the modified sequence regions. Consequently, more comprehensive strategies for covering adenoviruses have gained prominence. One innovative strategy involves using plasma-rich proteins such as albumin, to surround and shield the virus 20. This approach entails inserting an albumin-binding domain into the capsid protein of the adenovirus. Despite its novel concept, this strategy has limitations, primarily reduced infectivity caused by the insertion of the unnatural albumin-binding domain 20.

Thus, to preserve the natural character of adenovirus, the location of the sequence modification or domain insertion should be attentively designed with delicacy. The hypervariable regions (HVRs) of the adenovirus hexon protein may be an amenable region for modification, prompting extensive research into HVR modifications 21-23. Additional key considerations include ensuring the structural change of capsid by the inserted domain, abundancy of the targeted plasma binding protein, and the binding affinity. The transferrin binding domain from transferrin binding protein A (TbpA) of *Neisseria meningitidis* emerged as a suitable candidate, as TbpA binds transferrin regardless of iron binding status, unlike transferrin binding protein B (TbpB) 24. Our focus was thus on the transferrin binding domain of TbpA, particularly the loop 3 helix, which is critical for transferrin binding 25, 26. We adopted this domain and successfully inserted it into the HVRs.

This antibody-evading adenoviral vector system can be utilized for the treatment of metastatic cancers via systemic administration and also serve as an efficient tool for gene delivery.

## Results

### Insertion site determination of for exogenous peptide

**Figure 1:**
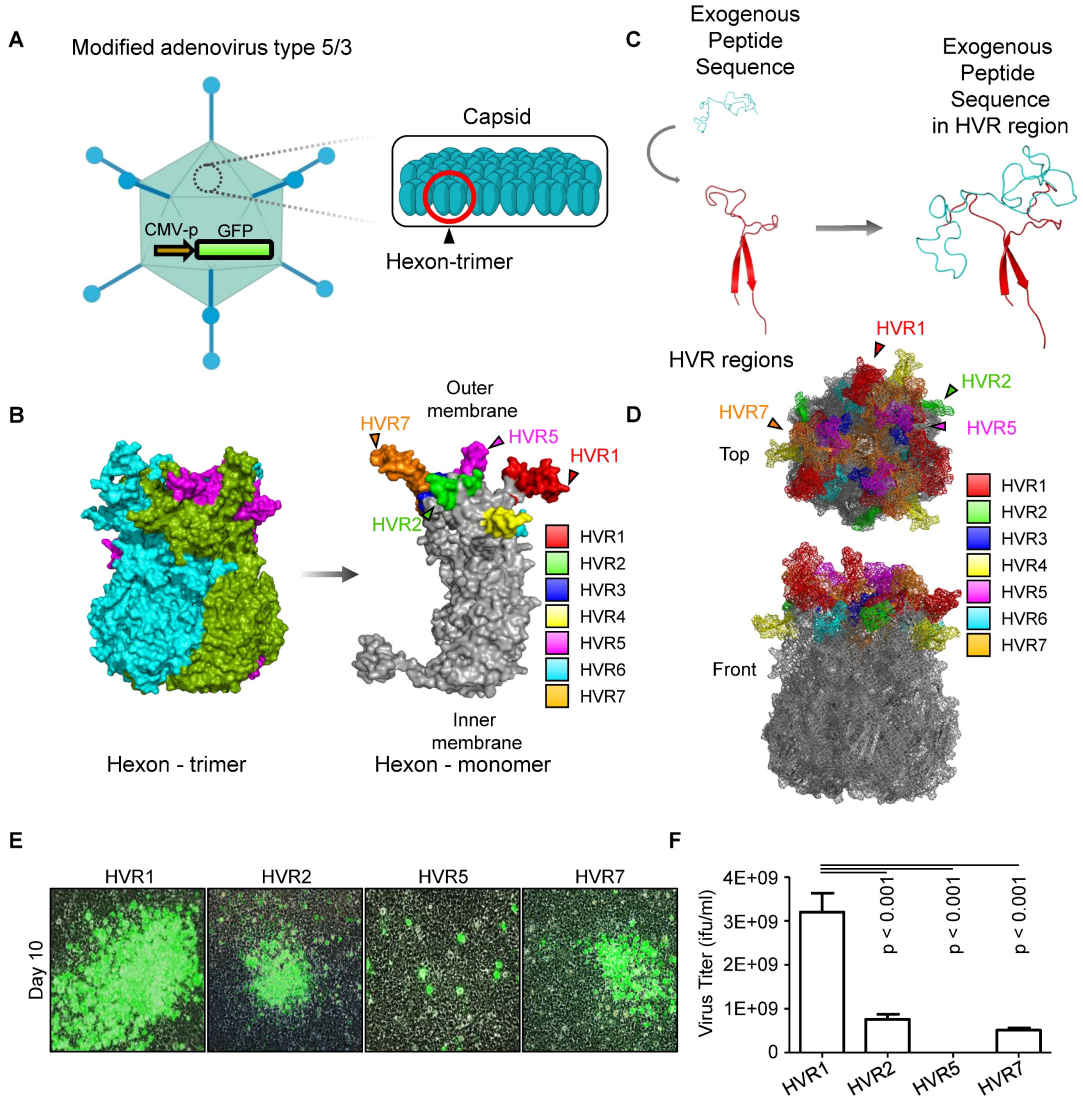
** Identification of hypervariable region (HVR) positioning for domain insertion within adenovirus type 5/3 hexon protein.** (**A**) The adenovirus type 5/3 utilized in this study was engineered to express green fluorescent protein (GFP). The hexon protein, a component of the adenovirus capsid, was targeted and modified in this research. (**B**) The structure of the hexon protein (both trimer and monomer forms), a component of the adenovirus type 5/3 capsid, was illustrated, highlighting the hypervariable regions 1-7 (HVR1-7) in color. (**C**) Conceptual illustration of exogenous peptide insertion into HVR domains. (**D**) Visualization of HVR domains within the hexon trimer. (**E** and **F**) The albumin binding domain (ABD) was inserted into the tip regions of HVR1, 2, 5, and 7. The productivity of ABD-inserted viruses at these positions was analyzed to identify the optimal insertion site. (**E**) Representative images of plaque formation in virus production. At 10 days post-transfection of plasmid vectors into HEK-293 cells, plaque formation was confirmed via imaging. (**F**) Lysates from the cells in E were obtained through three freeze-thaw cycles, and the virus titer of each lysate was calculated.

To establish a systemic injectable adenoviral vector, the optimal insertion site for exogenous peptides within the hexon protein needs to be experimentally determmined. Using a adenoviral vector, which is GFP-encoding oncolytic adenovirus serotype 5/3 (oAd5/3-GFP) having a basic backbone adenovirus, but GFP-expressed for visualization, we modified the hexon, unit protein of capsid (Figure 1A). Hexon protein exists as a trimer, with each monomer containing hypervariable regions 1-7 (HVR1-7), which are known to tolerate amino acid sequence modifications (Figure 1B). To evaluate spatial flexibility, an albumin-binding domain was used as the exogenous peptide first 27, 28, inserted into the bulged regions of each HVR domain (Figure 1C). Additionally, the HVRs (HVR1, 2, 5, and 7), which are the candidates for domain insertion, are indicated in the 3D hexon trimer model. (Figure 1D). We tested the remaining four HVRs for exogenous peptide insertion and measured the virus productivities (Figure 1, E and F). Viral replication-induced plaque was observed in HVR1, HVR2, and HVR7, but not in HVR5, with HVR1 showing a fourfold higher production yield compared to other sites.

**Figure 2:**
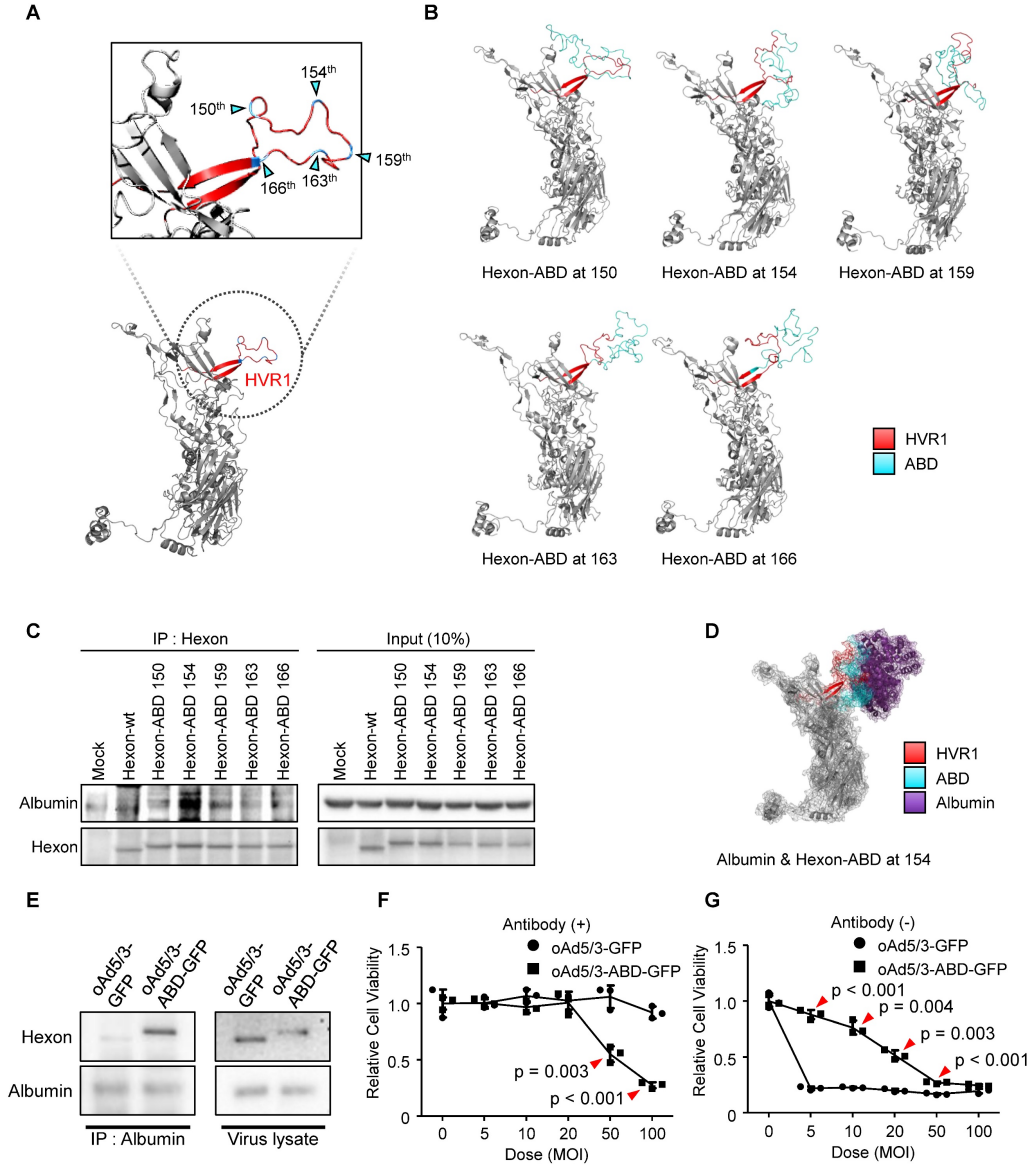
** Optimization for domain insertion position within hypervariable region 1 (HVR1).** (**A**) Protein structure of the adenovirus type 5/3 hexon. The magnified region shows HVR1 (red) with potential insertion sites for the albumin binding domain (ABD) marked by cyan triangles. (**B**) 3D-models of hexon proteins with ABD inserted at specific amino acid positions (150^th^, 154^th^, 159^th^, 163^th^, and 166^th^). (**C**) Comparison of albumin binding affinities of ABD-inserted hexon proteins using an immunoprecipitation assay. Vectors expressing either wild-type hexon or ABD-inserted hexon proteins were transfected into HEK-293 cells. Hexon proteins were over-expressed and pulled down with anti-adenovirus type 5/3 hexon antibody in the presence of albumin. (**D**) 3D-structure of the interaction between hexon-ABD (at the 154th position) and albumin protein. (**E**) Immunoprecipitation assay to confirm the albumin binding ability of the oncolytic adenovirus type 5/3 (oAd5/3-ABD-GFP) expressing GFP. 1.0 x 10^12^ viral particles were mixed with 2μg albumin protein and pulled down with an anti-albumin antibody. (**F**) Cell viability assay comparing the cytotoxic effects of oAd5/3-ABD-GFP and oAd5/3-GFP on 1.0 x 10^4^ A549 cells at various doses. (**G**) Cell viability assay assessing the antibody evasion capability of oAd5/3-ABD-GFP in the presence of 100ng/ml adenovirus neutralizing antibody on 1.0 x 10^4^ A549 cells at various doses.

To determine the optimal insertion site within HVR1 at the single amino acid resolution, five sites within its bulged region were selected (Figure 2A). The hexon proteins were designed to bind albumin protein by insertion of albumin binding domain (ABD) at each selected site (Figure 2B). Binding affinities between albumin and hexon were evaluated to identify the optimal site using immunoprecipitation method (Figure 2C). While hexons with insertions at positions 150, 159, 163, and 166 exhibited weak binding, the hexon with insertion at position 154 showed significantly higher binding affinity (Figure 2C), and 3D modeling of this binding interaction was conducted and analyzed. (Figure 2D). Using this optimized position, an ABD-inserted oncolytic adenovirus serotype 5/3 (oAd5/3) was produced (named oAd5/3-ABD-GFP), and binding was verified (Figure 2E). Although oAd5/3-ABD-GFP maintained an oncolytic effect even in the presence of blocking antibodies, unlike oAd5/3-GFP (Figure 2F). The oncolytic activity appeared to be reduced by ABD insertion (Figure 2G).

### Establishment of antibody-evading viral vector via transferrin binding feature

Albumin interacts with various biomolecules and drugs, affecting their pharmacological actions 29-31. This interaction may alter the concentration of active drugs, necessitating therapeutic drug monitoring in clinical settings 32, 33. Moreover, ABD insertion impaired viral infectious ability (Figure 2G), prompting the search for alternative domains that bind plasma proteins.

**Figure 3:**
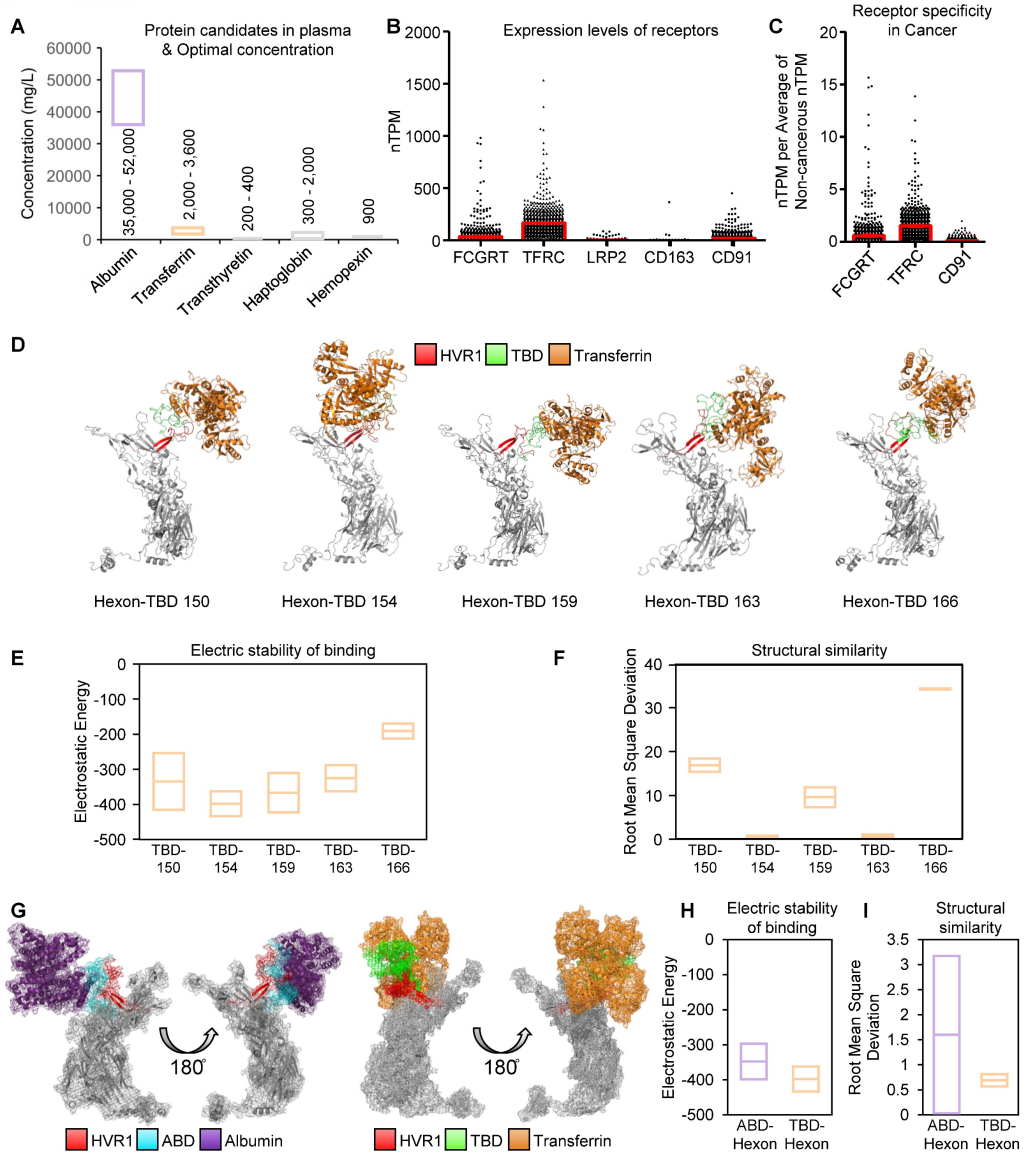
** Protein selection in human blood as a shielding protein for establishment of antibody-evading adenovirus.** (**A**) The blood plasma is enriched with five primary proteins. Low-optimal and high-optimal concentration in blood plasma were represented with box. (**B**) Bar plot with mean and SEM for expression levels of receptors for albumin using normalized transcripts per million (nTPM), transferrin, transthyretin, haptoglobin, and hemopexin across 1019 cancer cell lines. Receptors: FCGRT (albumin), TFRC (transferrin), LRP2 (transthyretin), CD163 (haptoglobin), and CD91 (hemopexin). (**C**) Comparison of receptor expression in cancerous vs. non-cancerous cells. nTPM of Figure 3B (n = 1019) were devided by average nTPM of non-cancerous cells (n = 63). Data was illustrated using bar plot with mean and SEM. (**D**) 3D-models of hexon proteins with TBD inserted at specific positions (150^th^, 154^th^, 159^th^, 163^th^, and 166^th^). (**E**) Analysis of binding stability for TBD-transferrin interaction using root mean square deviation (RMSD). Mean and standard-deviation were represented. (**F**) Analysis of binding stability for TBD-transferrin interaction using electrostatic energy. Mean and standard-deviation were represented. (**G**) Comparison of 3D-binding models of ABD-albumin and TBD-transferrin. (**H**) Comparison of TBD-transferrin and ABD-albumin binding stability using RMSD. Mean and standard-deviation were represented. (**I**) Comparison of TBD-transferrin and ABD-albumin binding stability using electrostatic energy. Mean and standard-deviation were represented.

Thus, we selected five plasma protein candidates to replace albumin, based on their concentrations in plasma 34 (Figure 3A). To evaluate potential cancer-specific delivery, receptor expressions for these plasma proteins were analyzed across 1019 human cancer cell lines using Human Protein Atlas 35-42 (Figure 3B). In addition, to exclude infection to normal cells we elicited cancer-specific receptors by the expression difference between cancerous- (n = 1019) and non-cancerous cells (n = 63) (Figure 3C). As a result, the transferrin receptor 1 (TFRC) emerged as the most highly expressed cancer-specific receptor.

To establish a transferrin binding domain (TBD), we adopted the transferrin binding motif from the *Neisseria* species' TbpA protein, which binds human transferrin independently of ferrous binding, unlike TbpB 24. The loop 3 helix of TbpA, critical for transferrin binding 25, 26, was investigated as a TBD. We optimized the insertion of TBD into oAd5/3 (named oAd5/3-TBD-GFP) by modeling interactions between modified hexon proteins and transferrin at multiple positions (150, 154, 159, 163, and 166) (Figure 3D). Molecular binding stability was assessed using electrostatic energy and root mean square deviation 43, 44 (Figure 3, E and F), with the 154th position showing the lowest scores and energy states, matching the optimal ABD insertion site. The binding stability of TBD-154 was the lowest one by electrostatic energy calculation (Figure 3E), and the TBD-154 model had the best structural similarity via the lowest value of root mean sqaure deviation (Figure 3F). 3D modeling revealed that TBD-transferrin binding was vertical, whereas ABD-albumin binding was horizontal, potentially causing interference between albumin molecules (Figure 3G). Binding affinities were predicted to be higher in TBD-inserted hexon with transferrin compared to ABD-inserted hexon with albumin (Figure 3, H and I).

### Validation of oAd5/3-TBD-GFP construction

**Figure 4:**
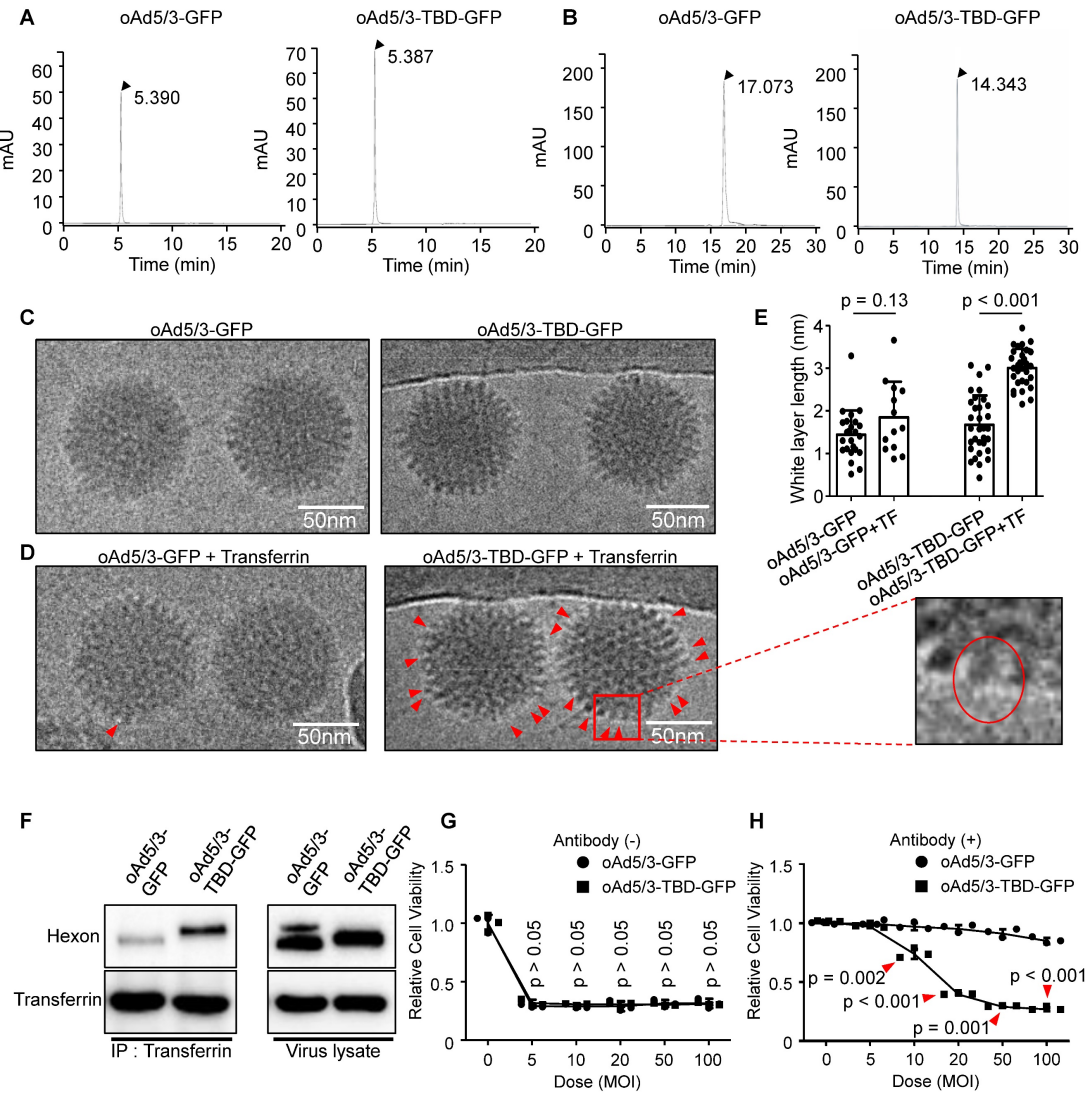
** Property validation of oAd5/3-TBD-GFP for construction.** (**A**) Size exclusion high-performance liquid chromatography (HPLC) analysis comparing the properties of oAd5/3-GFP and oAd5/3-TBD-GFP. (**B**) Ion exchange HPLC analysis comparing the properties of oAd5/3-GFP and oAd5/3-TBD-GFP. (**C**) Morphology of oAd5/3-GFP and oAd5/3-TBD-GFP visualized by cryo-EM. (**D**) Cryo-EM images of oAd5/3-GFP and oAd5/3-TBD-GFP after incubation with 1 μg/ml transferrin solution. Red arrows indicates white blob on the surface of virus. (**E**) The white layer size analysis of C and D conducted using ImageJ. (**F**) Immunoprecipitation assay to confirm transferrin binding ability of oAd5/3-TBD-GFP compared to oAd5/3-GFP. 1.0 x 10^12^ viral particles were mixed with 2μg transferrin protein and pulled down with an anti-transferrin antibody. (**G**) Cell viability assay comparing the cytotoxic effects of oAd5/3-TBD-GFP and oAd5/3-GFP on 1.0 x 10^4^A549 cells at various doses. (**H**) Cell viability assay assessing the antibody evasion capability of oAd5/3-TBD-GFP in the presence of adenovirus neutralizing antibody on 1.0 x 10^4^ A549 cells at various doses.

As illustrated in Figure 3, we developed oAd5/3-TBD-GFP, an adenovirus modified with a transferrin binding domain (TBD) inserted at the 154th position of the hexon protein. To check the physical properties of the engineered virus, a series of analyses were conducted. The size of oAd5/3-TBD-GFP was compared to that of oAd5/3-GFP using size exclusion-high-performance liquid chromatography (SEC-HPLC). The results indicated identical peak times for both, suggesting comparable sizes (Figure 4A). The surface charge properties, reflecting the outer membrane characteristics, were analyzed via ion exchange-high-performance liquid chromatography (IEC-HPLC). Unlike SEC-HPLC, the peak of oAd5/3-TBD-GFP appeared earlier than that of oAd5/3-GFP. This shift is attributable to the higher isoelectric point (relatively basic) of TBD compared to the viral hexon, which alters the elution profile (Figure 4B). According to the reference, the theoretical isoelectric points of the hexon and the TBD-inserted hexon were 5.17 and 5.25, respectively, while the isoelectric point of TBD alone was 8.06 45, 46. The peak shift observed in IEC-HPLC may be attributed to the exposure of the TBD on the outer membrane.

Further analysis involved visualizing virus establishment and transferrin binding using an electron microscope. The construction of oAd5/3-TBD-GFP was confirmed by its shape, which closely resembled that of oAd5/3-GFP (Figure 4C). The key acquired feature of oAd5/3-TBD-GFP was its ability to bind transferrin, as demonstrated by electron microscopy images showing transferrin binding (Figure 4D).

Since the non-tagged proteins appeared as white blobs 20, 47-49, the white layer was analyzed to assess the transferrin-virus interaction. Condensed white blobs were observed on the surface of oAd5/3-TBD-GFP, appearing to cover the virus, whereas no such layer was present on oAd5/3-GFP (Figure 4D). To quantify this interaction, the thickness of the white layer in Figure 4C and 4D was measured and analyzed (Figure 4E). In transferrin-incubated oAd5/3-GFP was similar in layer size to naïve oAd5/3-GFP, whereas transferrin-incubated oAd5/3-TBD-GFP showed an increase in layer size compared to naïve oAd5/3-TBD-GFP.

The interaction between oAd5/3-TBD-GFP and transferrin was re-validated through immunoprecipitation, confirming the virus's ability to evade antibodies via TBD-mediated shielding (Figure 4F). The oncolytic potential of oAd5/3-TBD-GFP remained unaffected by the TBD insertion (Figure 4G). Notably, in the presence of anti-adenovirus antibodies, oAd5/3-TBD-GFP sustained its oncolytic efficacy at multiplicities of infection (MOI) of 50 and 100 (Figure 4H).

### Property comparison of oAd5/3-TBD-GFP with oAd5/3-ABD-GFP

**Figure 5:**
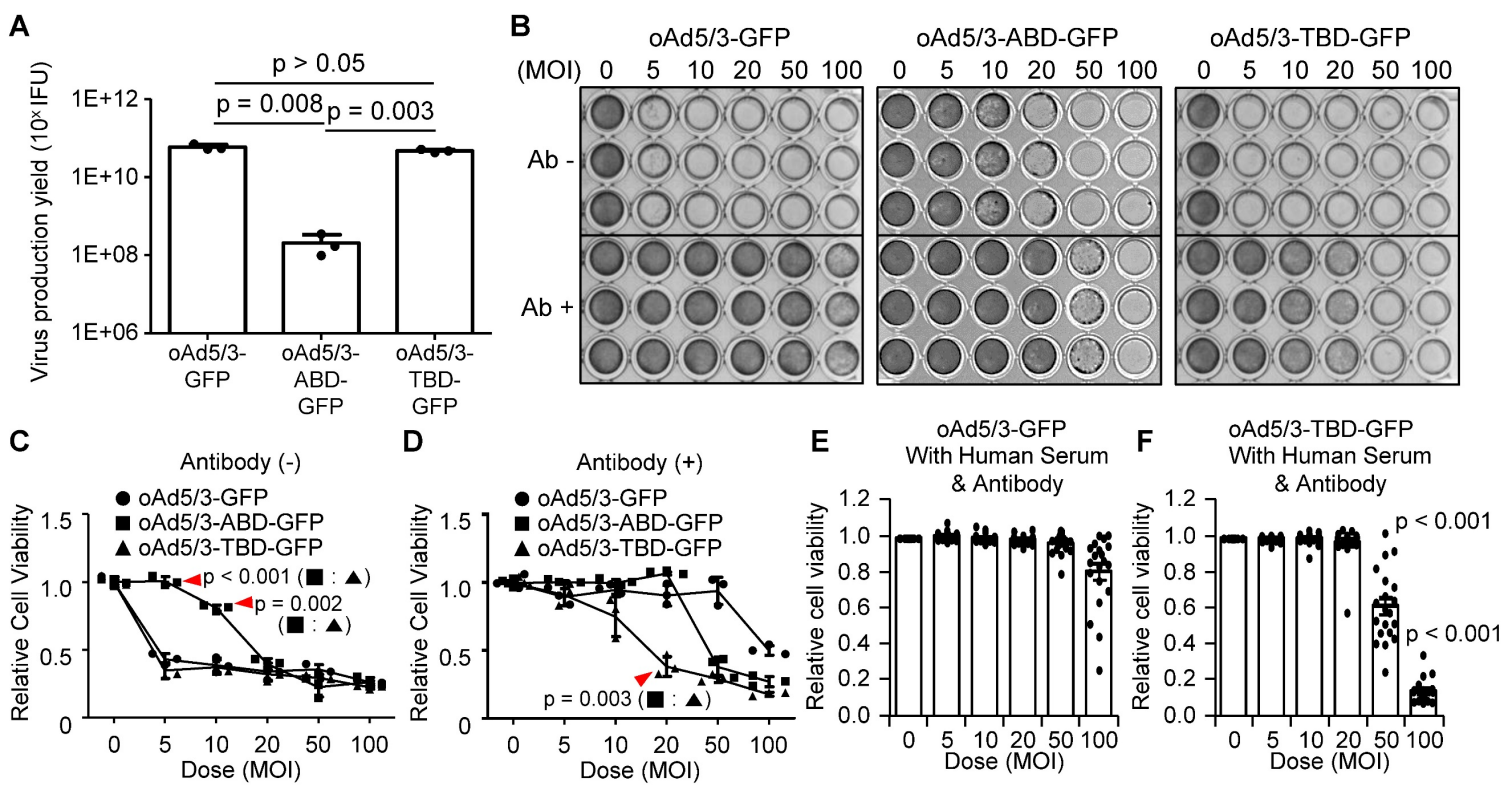
** Property comparison of oAd5/3-TBD-GFP with oAd5/3-ABD-GFP.** (**A**) Comparison of virus production yield between oAd5/3-GFP and oAd5/3-TBD-GFP. Viruses were produced in 1.0 x 10^9^ HEK-293 cells, harvested after 48 h, and quantified. (**B**) Comparison of the oncolytic abilities of oAd5/3-GFP, oAd5/3-ABD-GFP, and oAd5/3-TBD-GFP using crystal violet staining on A549 cells infected at different multiplicities of infection (MOI) with or without adenovirus neutralizing antibody. (**C** and **D**) Comparison of the oncolytic abilities of oAd5/3-GFP, oAd5/3-ABD-GFP, and oAd5/3-TBD-GFP using cell viability assays in the absence (**C**) and presence (**D**) of 50ng/ml of neutralizing antibody on A549 cells infected at different MOIs. (**E** and **F**) Cell viability tests evaluating the antibody-evading ability of oAd5/3-GFP (**E**) and oAd5/3-TBD-GFP (**F**) in RPMI medium containing 1% human blood serum and 100ng/ml anti-adenovirus neutralizing antibodies. The assay used serum samples from 20 individuals The statistical analysis was calculated between 50 MOI of (**E** and **F**), and 100 MOI of (**E** and **F**) using two-tailed t-test.

Finally to identify a clinically more useful antibody-evading virus, a comparative analysis was conducted between oAd5/3-ABD-GFP and oAd5/3-TBD-GFP. First, the yield of virus production was assessed. The productivity of oAd5/3-TBD-GFP was found to be 229 times higher than that of oAd5/3-ABD-GFP, indicating that ABD insertion partially impairs viral production (Figure 5A). Further, a comparison of viral cytotoxicity using crystal violet staining revealed that even though both viruses exhibited antibody-evading capabilities, the oncolytic capacity itself of oAd5/3-ABD-GFP was a little reduced compared with naïve oAd5/3 (Figure 5B). This finding was corroborated by cell viability assays (Figure 5, C and D). To evaluate the antibody-evading capability in human blood, 20 blood samples from healthy individuals were tested. The antibody evasion was assessed in 1% human serum media with added anti-adenovirus antibodies. In this setting, the infectivity of oAd5/3-GFP was entirely blocked across all doses (Figure 5E), whereas oAd5/3-TBD-GFP maintained its cytotoxicity (Figure 5F).

### Immune-refractory experiment of oAd5/3-TBD-GFP

Prior to initiating *in vivo* assessments of oAd5/3-TBD-GFP, the biodistribution profile of the viral construct was comprehensively analyzed. Spatiotemporal distribution studies demonstrated that intravenous administration of oAd5/3 in mice resulted in peak viral presence at the 24 h mark post-injection 50. Consistent with previous findings, we subsequently evaluated the biodistribution of oAd5/3-TBD-GFP at this time point (Figure S1). Comparative analyses indicated no statistically significant differences in organ distribution between oAd5/3-TBD-GFP and the parental vector, oAd5/3-GFP. Although natural clearance of the adenovirus occurred within 24 h post-injection 50, further confirmation was obtained by assessing the vectors' sensitivity to the anti-adenoviral agent cidofovir (Figure S2) 51-54. Both oAd5/3-TBD-GFP and oAd5/3-GFP exhibited comparable susceptibility to cidofovir, with no significant differences observed in their responsiveness.

**Figure 6:**
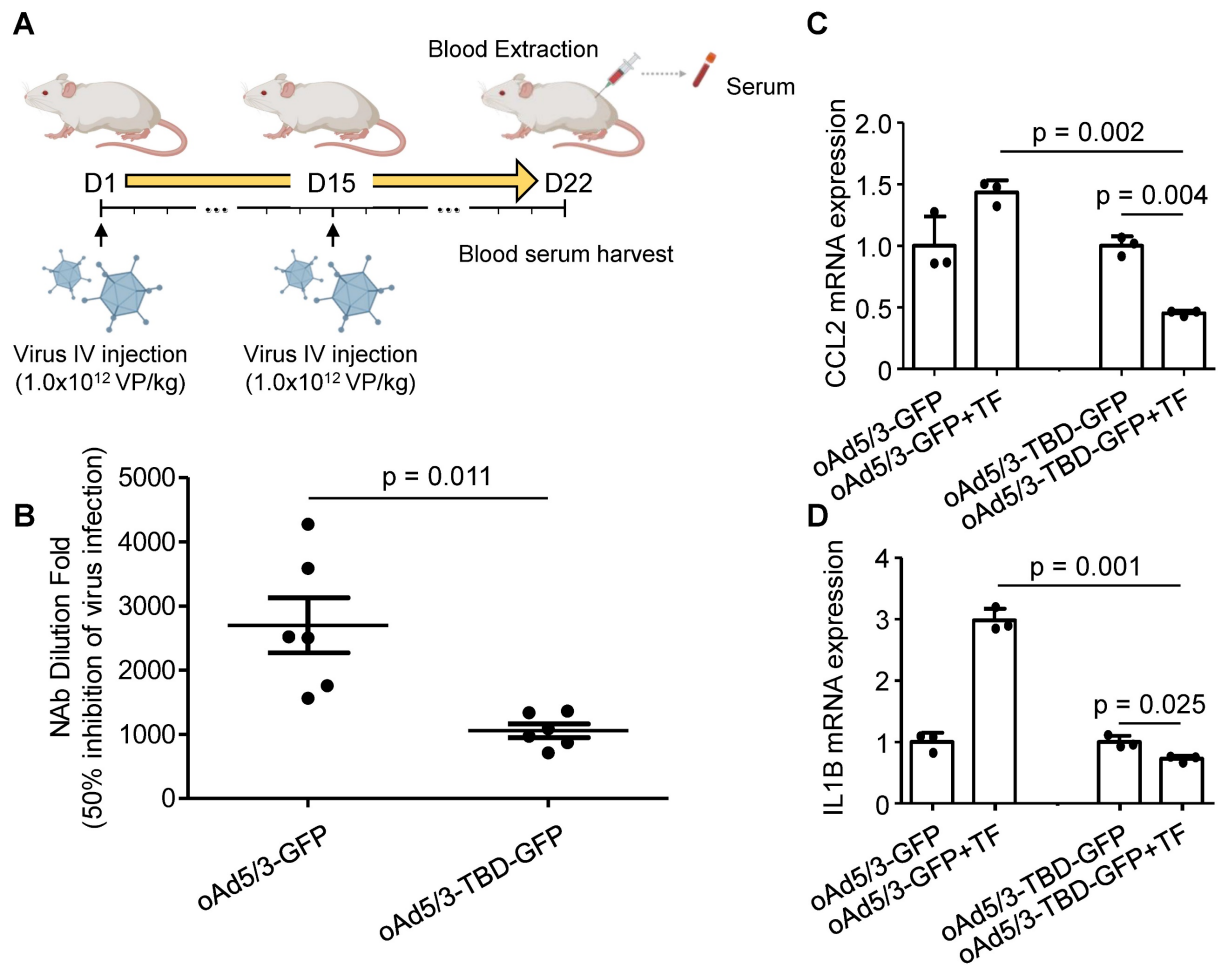
** oAd5/3-TBD-GFP evades recognition by the immune system *in vivo*.** (**A**) A schedule for harvesting blood serum samples from BALB/c mice exposed to oAd5/3-GFP and oAd5/3-TBD-GFP. (**B**) The dilution fold of mouse blood serum required to inhibit 50% of virus infection. The serum dilution fold for 50% inhibition of GFP expression is indicated in the table. (**C** and **D**) Differentiated U937 cells into M1 macrophages were infected with oAd5/3-GFP and oAd5/3-TBD-GFP, with or without 2μg/ml transferrin (TF). After 4 h, the expression of *CCL2* (**C**) and *IL-1B* (**D**) from M1 macrophages was analyzed.

To assess the immune response *in vivo*, we measured anti-adenovirus neutralizing antibody production. BALB/c mice were intravenously administered oAd5/3-GFP and oAd5/3-TBD-GFP on days 1 and 15. Blood samples were collected on day 22 (Figure 6A). Analysis of serum samples revealed that the antibody titer induced by oAd5/3-GFP was significantly higher, at 2.56 times that of oAd5/3-TBD-GFP (Figure 6B). Given that antibody production reflects immune system activation, the primary immune response was further investigated. M1 macrophages, known to recognize pathogens via phagocytosis and secrete inflammatory cytokines such as CCL2 and IL-1B, were studied using differentiated U937 cells. In the presence of transferrin, *CCL2* expression increased with oAd5/3-GFP but decreased with oAd5/3-TBD-GFP (Figure 6C). *IL-1B* expression mirrored the *CCL2* response (Figure 6D). Transferrin addition caused an increase in cytokine expression for oAd5/3-GFP. Whereas no such effect was observed with oAd5/3-TBD-GFP.

### Oncolytic efficacy of oAd5/3-TBD-GFP in a metastatic lung cancer model

**Figure 7:**
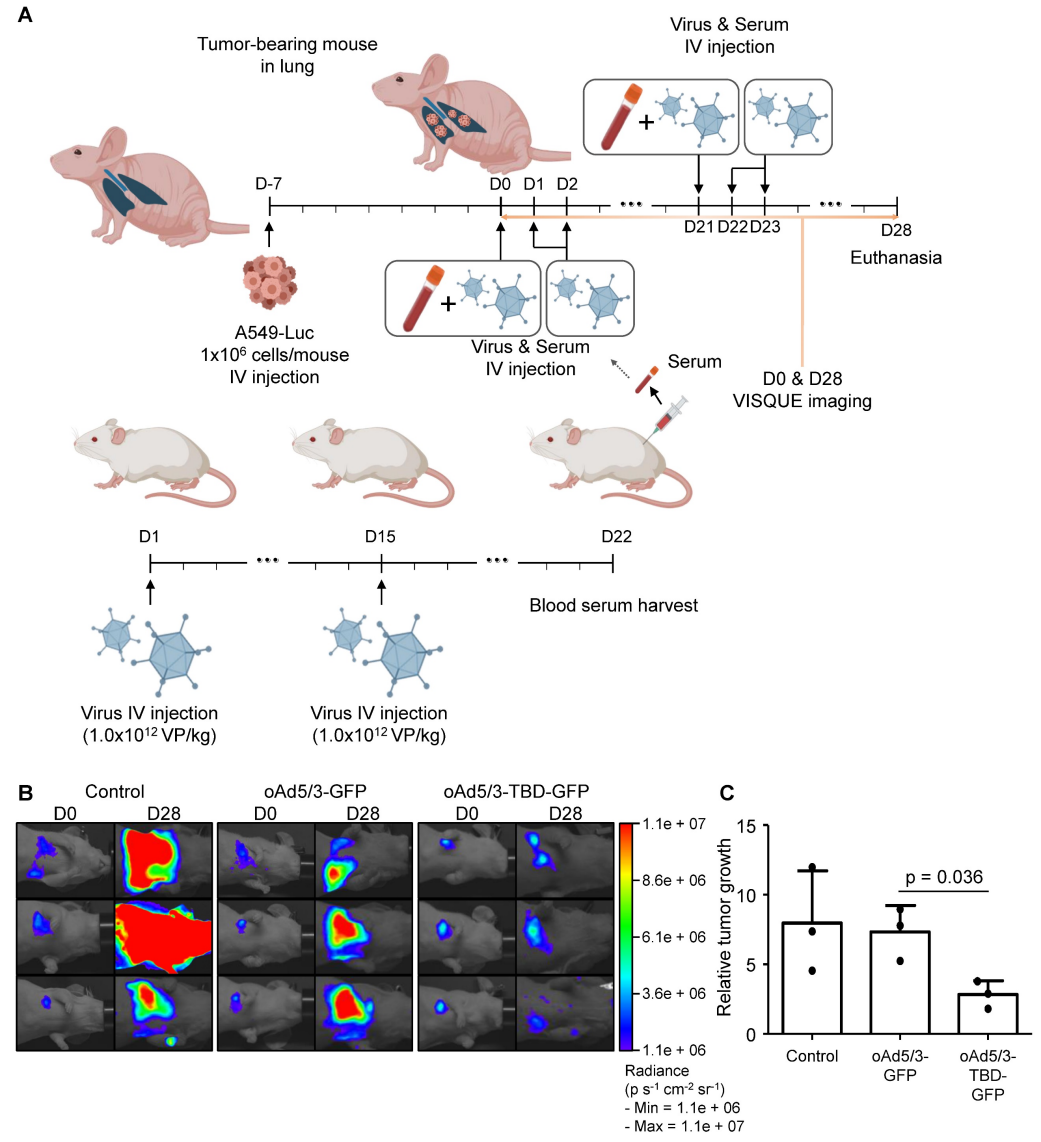
** oAd5/3-TBD-GFP maintains oncolytic effect in the presence of antibodies in a metastatic lung cancer model *in vivo*.** (**A**) A graphic illustrating the establishment of a metastatic lung cancer model with A549-luc cells and the production method for anti-adenovirus antibody from BALB/c mice (n = 3 per group). The administration route and schedule of A549-luc cells, virus, and serum are described. (**B**) Luciferase activities of A549-luc cells captured using a VISQUE instrument at 28 days after the first virus injection. (**C**) Graphs showing the calculation of A549-luc cancer cell growth based on VISQUE imaging and chemiluminescence. Tumor growth was normalized by calculating the ratio of intensity at day 28 (D28) relative to the baseline intensity at day 0 (D0) to minimize variability arising from differences in initial tumor size.

Prior to these *in vivo* studies, the antibody evasion potential of oAd5/3-TBD-GFP was assessed using mouse serum. The results confirmed that oAd5/3-TBD-GFP effectively circumvented antibody-mediated neutralization (Figure S3). To evaluate the oncolytic efficacy of oAd5/3-TBD-GFP, a metastatic lung cancer model was utilized to assess antibody evasion (Figure 7A). Tumor size was measured on day 28 using luciferase activity (Figure 7B). The oAd5/3-GFP treated group showed no significant regression in tumor growth. Conversely, the oAd5/3-TBD-GFP group exhibited significant tumor regression compared to oAd5/3-GFP treated groups. Tumor growth and statistical analysis are presented (Figure 7C). The therapeutic efficacy of oAd5/3-TBD-GFP was further investigated in a metastatic ovarian cancer model (Figure S4). The model was established via intraperitoneal injection of cancer cells, followed by intravenous administration of the virus. Tumor burden was monitored through bioluminescent imaging (Figure S4A). Notably, treatment with oAd5/3-TBD-GFP resulted in a substantial reduction in tumor growth, whereas the oAd5/3-GFP-treated group showed tumor progression akin to the control group (Figure S4B).

### CD8^+^ T cell infiltration enhanced by oAd5/3-TBD-GFP through antibody evasion

**Figure 8:**
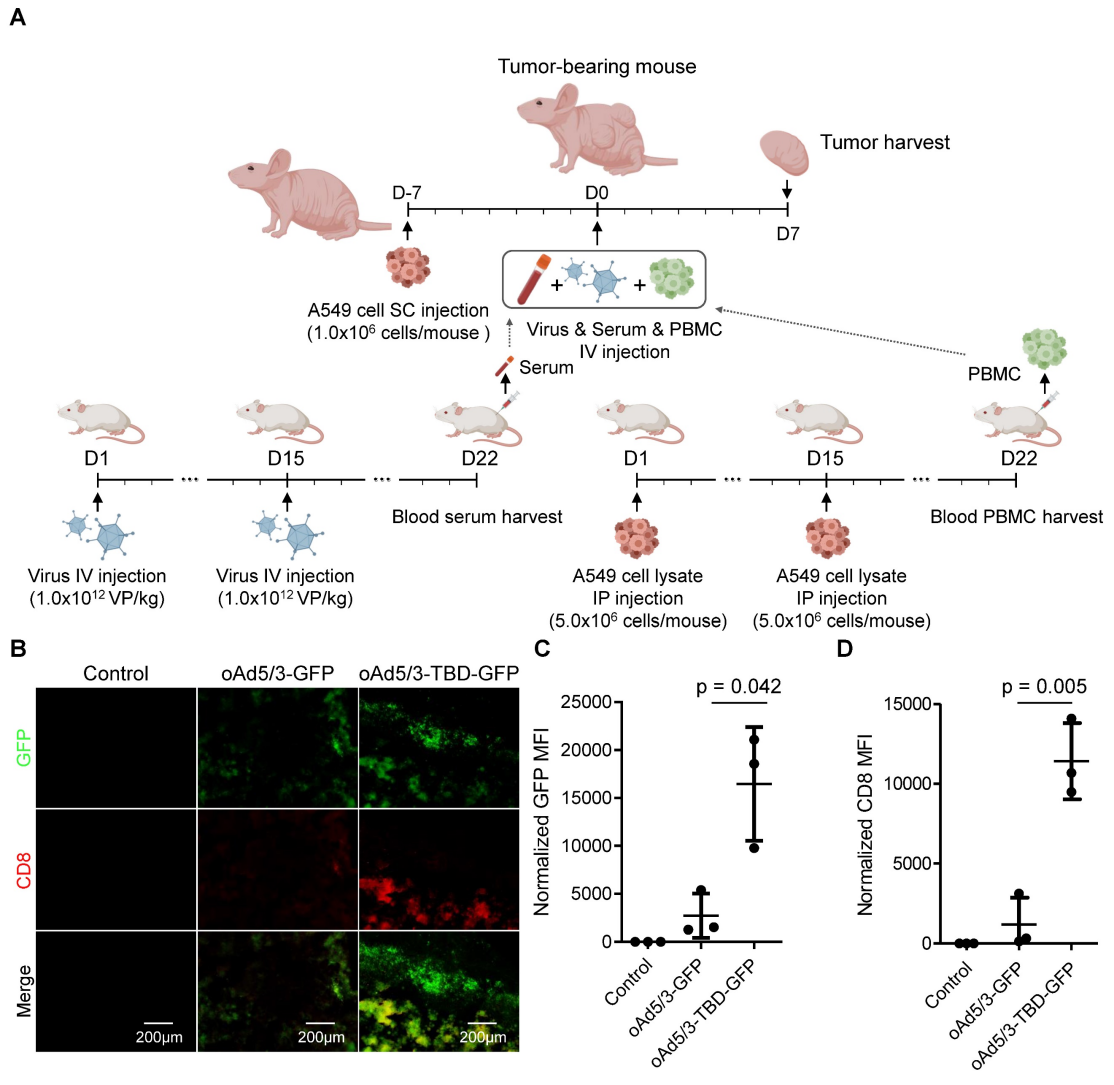
** CD8^+^ T cell infiltration is augmented in the oAd5/3-TBP-GDP treatment group in a xenograft mouse model by increased infection through antibody evasion.** (**A**) A graphic illustrating the establishment of a lung cancer xenograft mouse model with A549 cells and the production method for anti-adenovirus antibody and A549-adapted PBMC from BALB/c mice. The administration route and schedule of A549 cells, virus, serum, and PBMC are described (n = 3 per group). (**B**) Representative images of GFP and CD8 expression in tumors from (**A**). Tumors were harvested and sectioned on days 7 to analyze virus infection and CD8^+^ T cell infiltration. Tumor sections were stained with CD8 antibody (red), and fluorescence images were captured, showing green for virus infection and red for CD8^+^ T cell infiltration. (**C**) Quantification of GFP expression from (**B**). (**D**) Quantification of CD8 expression from (**B**).

To further explore the immune response, we examined tumor-infiltrating CD8^+^ T cell populations. Serum containing specific antibodies against oAd5/3-GFP or oAd5/3-TBD-GFP, along with adapted peripheral blood mononuclear cells (PBMCs), were transplanted into a tumor-bearing nude mouse model (Figure 8A). On day 7, tumors were harvested and stained for CD8^+^ T cell markers. Both virus infection and CD8^+^ T cell presence were detected in the tumors of both treatment groups (Figure 8B). Expectedly, the levels of infection and CD8^+^ T cell recruitment were significantly higher in the oAd5/3-TBD-GFP group compared to the control (Figure 8, C and D). In contrast, there were no significant differences observed in CD4^+^ T cell recruitment between groups (Figure S5A-C).

## Discussion

Despite recent success in developing effective adenovirus-based oncolytic viruses 2, 4, upon systemic administration, the primary obstacle to the efficacy of the viral therapy has been the host immune response, particularly antibody recognition. The recent clinical trial, despite its noted advancements, continued to encounter challenges related to the immune response 55. Due to the increase in neutralizing anti-adenovirus antibodies, the efficacy of the treatment might be compromised. Although adenovirus offers a safer profile compared to other viral vectors, the prevalence of anti-adenoviral antibodies is significantly higher 12-14. This immune recognition hampers the delivery of intravenously administered adenoviruses to tumor cells, as they are often neutralized before reaching their target. For these reasons, several studies are focused on the bladder, which allows for more efficient non-systemic delivery to evade systemic immune reactions 2, 50. To overcome this limitation, we engineered a novel adenovirus capable of infecting cells in the presence of antibodies through its interaction with human blood transferrin (Figure 5F).

In our research, adenovirus serotype 5/3 (oAd5/3), where the knob is replaced with that of adenovirus serotype 3 to enhance gene delivery and antitumor efficacy, was utilized as a basic backbone for modification 56-63. The results related to virus neutralization indicated that the traditional oAd5/3 (oAd5/3-GFP) lost its infectivity in the presence of anti-adenovirus antibodies, resulting in reduced efficacy both *in vitro* and *in vivo*. However, oAd5/3-TBD-GFP successfully evaded attacks of antibody, including those from the innate immune system.

The concept of antibody-evading oAd5/3-TBD-GFP involves covering the virus with transferrin protein. When transferrin proteins coat oAd5/3-TBD-GFP, the innate immune system's recognition of the virus as a foreign antigen should be reduced. This theoretical process was validated by the decreased recognition by M1 macrophages (Figure 6, C and D). Sequentially, the innate immune system's antigen recognition leads to antibody production for the antigen, which was also observed to decrease (Figure 6, A and B). The basic concept of transferrin interaction aims to evade antibody attacks from both pre-existing antibodies (Figure 5, C-F, Figure 7B, and Figure 8B) and newly produced antibodies (Figure 6B). This immuno-silencing effect is thus demonstrated from a multi-dimensional perspective.

The previously developed albumin-binding domain (ABD)-based virus faced significant limitations for systemic delivery, including reduced infectivity and unpredictable interactions due to the high concentration and diverse nature of albumin (Figure 2G). In contrast, oAd5/3-TBD-GFP did not exhibit such reductions in infectivity (Figure 5, B and C) 20. Additionally, oAd5/3-TBD-GFP demonstrated the ability to evade not only antibody-mediated neutralization but also broader immune recognition (Figure 6 and Figure 7), with a concomitant increase in CD8^+^ T cell recruitment (Figure 8).

As the oncolytic virus has the potential as a combinatorial regimen with immune checkpoint inhibitors, the challenge of oncolytic adenoviral therapy lies in balancing immune evasion and immune activation. The goal is to evade immunosurveillance during systemic delivery while enhancing immune response at the tumor site to maximize anti-cancer effects through immune cell recruitment. The engineered virus oAd5/3-TBD-GFP seemed to successfully achieve this balance. It evaded antigen recognition and antibody attacks during systemic circulation (Figure 5, Figure 6, and Figure 7) and promoted CD8^+^ T cell recruitment at the tumor site (Figure 8). Given that immune checkpoint inhibitors (ICIs) enhance the anti-cancer activity of CD8^+^ T cells, oAd5/3-TBD-GFP emerges as a promising combinatory partner for ICIs, providing a targeted approach to cancer therapy.

Recent advancements have concentrated on cytokine-armed oncolytic viruses, such as those encoding granulocyte-macrophage colony-stimulating factor (GM-CSF) and interferon-α 1, 2. While these viruses demonstrate significant efficacy in specific indications, their ability to treat a broad range of cancer types remains limited, necessitating systemic administration. Thus, the development of a systemically injectable virus, like our oAd5/3-TBD-GFP, represents a significant leap forward. This virus not only functions as an oncolytic agent but also serves as a versatile gene therapy vector, capable of delivering a wide array of genetic materials.

Transferrin was chosen over albumin due to its lower serum concentration, which is sufficient to shield oAd5/3-TBD-GFP. A single viral particle of oAd5/3-TBD-GFP, comprising 720 hexon proteins, requires only 2μg of transferrin to coat 1 x 10^12^ viral particles, within the available transferrin concentration of 2,000-3,600μg/mL in blood. Moreover, leveraging transferrin receptor-mediated delivery pathways, as evidenced in brain delivery and cancer targeting studies 64, 65, suggests that oAd5/3-TBD-GFP may enhance tumor selectivity.

Taken together, the optimized insertion of the transferrin-binding domain into adenovirus may provide a new aspect to gene delivery expecting subsequent clinical implications.

## Conclusion

The systemic injectable oncolytic adenovirus oAd5/3-TBD-GFP, engineered to evade antibody-mediated neutralization through the insertion of a transferrin-binding domain at the HVR1-154 position, was successfully developed. The antibody evasion capability of oAd5/3-TBD-GFP was validated both *in vitro* and *in vivo*. Additionally, oAd5/3-TBD-GFP exhibited reduced immunogenicity and enhanced tumor infiltration by CD8^+^ T cells, resulting in significant tumor size reduction in a metastatic lung cancer mouse model.

## Methods

### Production of oncolytic adenoviruses

The oncolytic adenovirus serotype 5/3 (oAd5/3)-GFP, oAd5/3-ABD-GFP, and oAd5/3-TBD-GFP were generated using adenovirus-producing plasmid vectors obtained from O.D.260 Inc. To ensure cancer cell-specific replication, the E1 promoter region was replaced with the human telomerase reverse transcriptase (hTERT) promoter for both oAd5/3-GFP and oAd5/3-TBD-GFP. For GFP expression, CMV promoter was used, and it is graphically described in Figure 1A. For the specific features of oAd5/3-TBD-GFP, the native hexon gene was substituted with a hexon gene bearing the transferrin binding domain. Albumin binding domain was following amino acid sequence; MGCSSHEHEHEDEAVDANSLAAAKETAL-YHLDRLGVADAYKDLIDKAKTVEGVKARYFEILHALPDDNEDEVDEQAEQQKTHVFGQA. Transferrin binding domain was following amino acid sequence; MDMTVPAFLTKAVFDANKKQAGSLPGNGKYAGNHKYGGLFTNGENGALVGAEYGT. These domains were incorporated at multiple insertion sites as illustrated in Figures 1-3, with the primary insertion point for the transferrin-binding domain (TBD) and albumin-binding domain (ABD) identified at the 154^th^ amino acid position of the hexon protein.

### Quantitative analysis of adenovirus titer

The concentration of adenovirus was determined using the infectious unit (IFU), calculated based on the ratio of adenovirus-infected cells to total cells in the field of view. HEK-293 (KCLB, 21573) cells were seeded into a 12-well plate at a density of 5×10^5^ cells per each well. The adenovirus solution was serially diluted from 1:10^1^ to 1:10^9^. After 48 h of incubation with the virus diluent, cells were washed with PBS and fixed with -20℃ methanol. The concentration of adenovirus was then analyzed using the Adeno-X™ Rapid Titer Kit (Takara, 632250).

### Receptor Expression analysis in cancerous and non-cancerous cell lines

Receptor expression levels across various cancerous and non-cancerous cell lines were obtained from The Human Protein Atlas and visualized using GraphPad Prism.

### 3D-structure modeling and binding affinity calculation

Following the incorporation of the domain into the hexon gene sequence, the corresponding amino acid sequence was used to generate a 3D structural model via the SWISS-MODEL server 66. To evaluate protein-protein binding affinities, the 3D structures of the domain-modified hexon protein, albumin, and transferrin were obtained in PDB format. These structures were then uploaded into HADDOCK to calculate binding affinities and generate interaction models 44. Visualization of protein structures was performed using PyMOL.

### Cryogenic electron microscopy (Cryo-EM)

A 4 μL aliquot of virus sample, diluted in PBS, was applied to a hydrophilic grid (Quantifoil, R1.2/1.3, 300 mesh, EMS) prepared using a glow discharge system (PELCO easiGlow^™^, Ted Pella). The sample was blotted for 1.5 seconds at 4°C with 100% humidity, using a force setting of -3. Following vitrification in liquid ethane (Vitrobot Mark IV, FEI), the samples were analyzed at 120 kV using an electron microscope (Talos L120C, FEI).

### Immunoprecipitation assay

2μg of transferrin (InVitria, 777TRF029) or 2μg albumin (Sigma, A1653) was added to 1.0 x 10^12^ viral particles of oAd5/3-GFP and oAd5/3-TBD-GFP (or oAd5/3-ABD-GFP), and the mixtures were incubated at room temperature for 2 h. For pull-down, 2μg of anti-transferrin antibody (Santa Cruz, sc-365871) or 2μg of anti-albimin antibody (Santa Cruz, sc-271605) was added to each tube, and protein was collected using protein A/G agarose beads (Santa Cruz, sc-2003).

### Immunoblotting assay

5x SDS sample buffer (LPS solutions, CBS002) and 1x cell lysis buffer (Cell Signaling, 9803S) were utilized for SDS-PAGE. All samples were loaded onto SDS-polyacrylamide gels and electrophoresed at 80 V for 30 min and then at 120 V for 90 min. Proteins were transferred to polyvinylidene fluoride (PVDF) membranes (Merck, IPVH08100). The PVDF membranes were blocked with blocking buffer which is 5% skim milk in TBST buffer (LPS solutions, CBT007L). Primary antibody incubation was performed at a dilution fold of 1:1,000, followed by secondary antibody incubation at a dilution of 1:10,000, both in blocking buffer. All antibodies were diluted accordingly.

### Antibody evasion ability test for oAd5/3-ABD-GFP or oAd5/3-TBD-GFP

To coat the virus with a coating protein, viruses were incubated in a 2μg/ml albumin solution or 2μg/ml transferrin solution state for 1 h at 4℃. The coated virus was then administered to cells, and antibodies were simultaneously diluted into the cell growth medium at a 1:1,000 dilution fold. In this experiment, anti-adenovirus antibodies (Abcam, ab6982) were used as the adenovirus neutralizing reagent.

For the method using human blood serum, blood samples were obtained from twenty voluntary blood donors (IRB no. KHUH2023-01-016-001). The blood samples were centrifuged at 1,500xg for 20 min, and serum samples were harvested. For the experiment on transferrin-mediated antibody evasion on the virus, blood serum containing transferrin was used instead of recombinant transferrin solution.

### Cell viability assay

Cell viability was measured by trypan blue staining. Cells were harvested with 0.05% trypsin-EDTA solution after appropriate treatment. Harvested cells were stained with trypan blue solution (ThermoFisher, T10282) for 5 min, and the proportion of live and dead cells was measured automatically using the Countess 3 instrument (ThermoFisher, AMQAX2000). Each batch was measured three times.

### Crystal violet staining

Cells were fixed with pre-chilled 100% methanol for 5 min at -20°C. Subsequently, a 1% crystal violet solution (Sigma, V5265) was added to the cells. After methanol fixation and crystal violet staining, cells were washed three times with PBS.

### Purity confirmation of adenovirus through size exclusion-high-performance liquid chromatography (SEC-HPLC) and ion exchange-high-performance liquid chromatography (IEC-HPLC)

SEC-HPLC was performed using a 1290 Infinity II Prime HPLC (Agilent) and a TSKgel® G3000SWXL HPLC Column (MERCK). PBS was used for priming and washing steps. For IEC-HPLC, a Resource™ Q column (Cytiva) was utilized. Trizma-based buffer was used for priming and washing steps.

### Animal experiments

Five-week-old BALB/c male mice and five-week-old BALB/c nude male mice were purchased from Orient Bio (Gyeonggi, Korea). All animal experiments were reviewed and approved by the Institutional Review Board (IRB, approval number: KHSASP-24-117) and the Institutional Animal Care and Use Committee (IACUC, approval number: CRG-RNDC02.01-02), and performed according to the criteria of the IRB and IACUC guidelines. Mice were maintained in pathogen-free facilities.

### Assessment of neutralizing antibody titers in mouse serum

To determine the efficacy of neutralizing antibodies, 1.0 × 10⁴ A549 cells were seeded into 96-well plates. Serial 1:10 dilutions of mouse serum were prepared, and cells were infected with either oAd5/3-GFP or oAd5/3-TBD-GFP in the presence of the corresponding neutralizing serum. After 24 h, neutralizing antibody titers were quantified using the Fluoroskan™ FL Microplate Fluorometer (ThermoFisher). A standard curve was established using a commercial anti-adenovirus neutralizing antibody (Abcam, ab6982), which demonstrated that a 1:40,000 dilution inhibited 50% GFP expression for both viral constructs. Neutralizing antibody titers were then calculated based on this standard curve.

### Antibody productivity test *in vivo*

For the antibody productivity test, 1.0 x 10^12^ viral particles (VP)/kg of oAd5/3-GFP and oAd5/3-TBD-GFP were injected intravenously into six-week-old BALB/c male mice on day 1 (n = 6 per group). To boost antibody production, 1.0 x 10^12^ VP/kg of oAd5/3-GFP and oAd5/3-TBD-GFP were reinjected intravenously on day 15. On day 22, all mice were euthanized, and blood was harvested.

### Metastatic lung and ovarian cancer model and *in vivo* neutralization assay

To immunize BALB/c nude mice against human cells, materials derived from BALB/c mice were injected into BALB/c nude mice. 1.0 x 10^12^ VP/kg were injected into male BALB/c mice on day 1 through the tail vein. To boost antibody production, 1.0 x 10^12^ VP/kg were reinjected into male BALB/c mice on day 15 through the same injection point as before. On day 22, mice were euthanized, and serum was extracted from blood samples (n = 6). Each serum sample was mixed and titrated using the neutralizing antibody assay protocol.

For the metastatic lung cancer model, 1.0 x 10^6^ A549-luc2 cells (ATCC) were injected intravenously into six-week-old BALB/c nude male mice (n = 3 per group) via tail vein. After 7 days (on day 0), a 20 μL mixture of serum, equivalent in potency to 10 μg of anti-adenovirus antibody (Abcam, ab6982), was administered via tail vein injections on days 0 and 21, immediately prior to viral administration. On days 0, 1, 2, 21, 22, and 23, 5 x 10^8^ ifu of oAd5/3-GFP and oAd5/3-TBD-GFP were intravenously injected into the mice through tail vein.

For the metastatic ovarian cancer model, 1.0 × 10⁵ HeyA8-luc cells (kindly provided by Prof. Jung-Won Lee, Samsung Medical Center, Korea) were intraperitoneally injected into six-week-old BALB/c nude female mice (n = 3 per group) on day 0. On day 4, 20 µL of serum with neutralizing potency equivalent to 10 µg of anti-adenovirus antibody (Abcam, ab6982) was administered intravenouslly. Concurrently, mice received an intravenous injection of 5 × 10⁸ IFU of either oAd5/3-GFP or oAd5/3-TBD-GFP.

### Bioluminescence imaging

For bioluminescence imaging, 3mg of luciferin (Merck, L6152) was intraperitoneally injected into mice. After 10 min, the mice were placed into the VISQUE InVivo Smart-LF instrument (VIEWWORKS, BI24001). Luciferase activity was then analyzed in units of radiance (p s^-1^ cm^-2^ sr^-1^).

### Establishment of mouse model of CD4^+^ and CD8^+^ T cell infiltration

To induce immunity against A549 cells in BALB/c mice, 5.0 x 10^6^ A549 cells, a human alveolar adenocarcinoma cell line, were injected into mice intraperitoneally on days 1 and 15. On day 22, mice were euthanized, and peripheral blood mononuclear cells (PBMCs) were isolated from blood samples. Immediately after PBMC isolation, 5.0 x 10^6^ PBMCs were intravenously injected into BALB/c nude mice as a supply of T cell immunity on day 0 of the tumor-bearing nude mouse model. The protocol used in this article for PBMC transplatation is developted based on *in vitro* activated T cell transplantation protocol 67-74.

Subsequently, 1.0 x 10^6^ A549 cells (KCLB, 10185) were subcutaneously xenografted into six-week-old male BALB/c nude mice. Seven days after A549 cell injection, 20μl of blood serum which is mentioned at establishment of metastatic lung cancer mouse model in method section, 5.0 x 10^6^ PBMCs, and 5.0 x 10^8^ ifu of viruses (oAd5/3-GFP and oAd5/3-TBD-GFP, with an equivalent volume of PBS used for the virus control group) were intravenously injected into the tumor-bearing BALB/c nude mouse model (n = 3 per group). At 7 days after the administrations, tumor-bearing nude mice were euthanized, and tumors were harvested and snap-frozen for sectioning.

### Immunofluorescence staining

To analyze the expression of CD4 and CD8 in tumors, samples were sectioned and stained for the analysis of target proteins. Tumor samples were placed on slide-glass and fixed with -20℃ methanol for 10 min. Subsequently, a 5% bovine serum albumin solution in PBS was used for the blocking step. CD4 and CD8 antigen was stained using Alexa Fluor 546-conjugated anti-CD4 antibody (Santa Cruz, sc-19641 AF546) and Alexa Fluor 546-conjugated anti-CD8 antibody (Santa Cruz, sc-1177 AF546).

### Statistical analysis

The statistical significance of the data was determined using a two-tailed t-test. Significance levels are indicated in each figure, with the compared groups marked by bars under the respective p-values.

## Supplementary Material

Supplementary figures.

## Abbreviations

FDA: food and drug administration
T-VEC: talimogene laherparepvec
HVRs: hypervariable regions
TbpA: transferrin binding protein A
TbpB: transferrin binding protein B
oAd5/3: oncolytic adenovirus serotype 5/3
GFP: green fluorescent protein
ABD: albumin binding domain
FCGRT: Fc gamma receptor and transporter
TFRC: transferrin receptor 1
LRP2: low-density lipoprotein (LDL) receptor related protein 2
TBD: transferrin binding domain
SEC-HPLC: size exclusion-high-performance liquid chromatography
IEC-HPLC: ion exchange-high-performance liquid chromatography
MOI: multiplicities of infection
CCL2: C-C motif chemokine ligand 2
IL-1B: interleukin 1 beta
PBMCs: peripheral blood mononuclear cells
ICIs: immune checkpoint inhibitors
GM-CSF: granulocyte-macrophage colony-stimulating factor
hTERT: the human telomerase reverse transcriptase
IFU: infectious unit
PBS: phosphate buffered saline
Cryo-EM: cryogenic electron microscopy
SDS-PAGE: sodium dodecyl sulfate polyacrylamide gel electrophoresis
PVDF: polyvinylidene fluoride
TBST: tris buffered saline with tween-20
EDTA: ethylene-diamine-tetraacetic acid
VP: viral particle
