# Supplementary material for: Transferrin-binding domain inserted-adenovirus hexon engineering enables systemic immune evasion and intratumoral T-cell activation
Source: Theranostics. 2025 Jan 1;15(4):1221–37. doi: 10.7150/thno.105163 (PMC11729563; doi:10.7150/thno.105163)
Supplement: Supplementary file 1 — Supplementary figures. [file thnov15p1221s1.pdf]

Supplementary data

Title: Transferrin-binding domain inserted-adenovirus hexon engineering enables systemic immune evasion and intratumoral T-cell activation

Author(s): Dae Hoon Lee, Youngtae Kwon, Ki Hwan Um, Jung Ki Yoo, Wootae Ha, Ki-Su Kim, Jintak Cha, Ha-Eun Cho, Kyung Sun Park, Min Jeong Kye, Jin Woo Choi

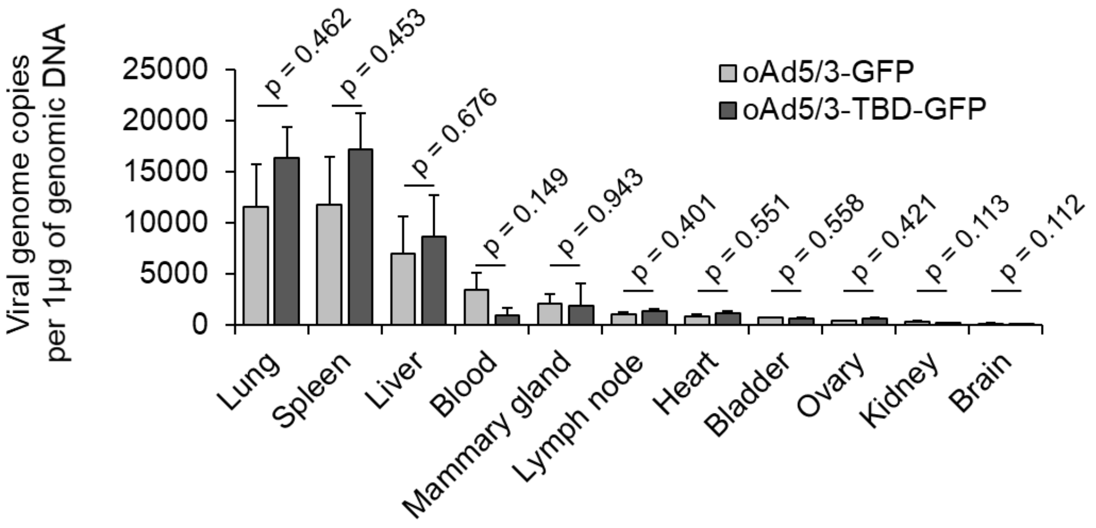

Figure S1. Comparative organ biodistribution of oAd5/3-GFP and oAd5/3-TBD-GFP in a mouse model.

Mice (n = 3 per group, 6 weeks old) were intravenously administered with 5.0×10^9 infectious units (IFU) per kg of either oAd5/3-GFP or oAd5/3-TBD-GFP. At 24 h post-injection, all animals were euthanized, and their organs were harvested for viral biodistribution analysis. Genomic DNA was extracted from each organ, and viral load was quantified by determining the copies of the viral genome.

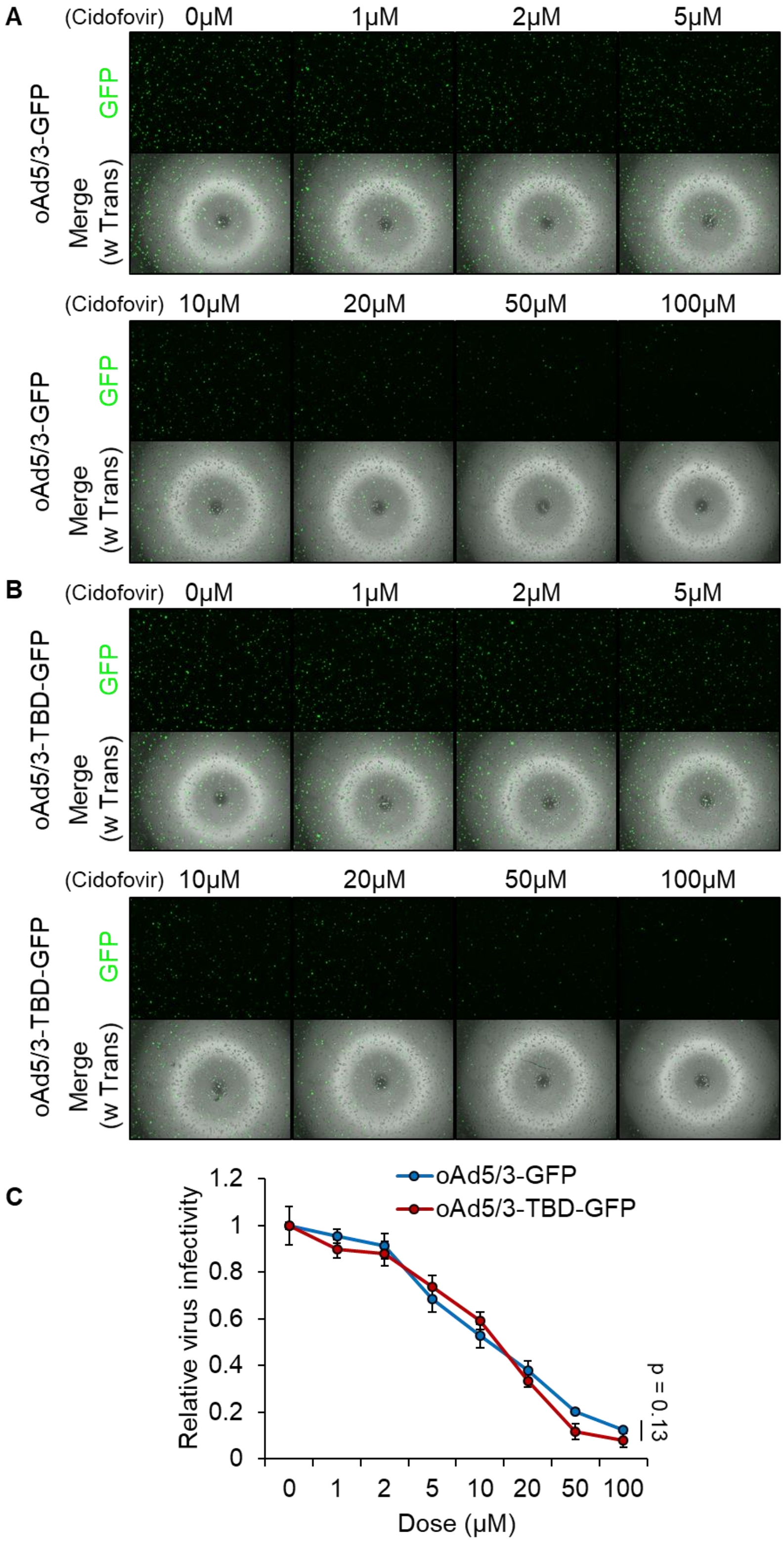
A
(Cidofovir) 0μM 1μM 2μM 5μM
oAd5/3-GFP
GFP
Merge (w Trans)
(Cidofovir) 10μM 20μM 50μM 100μM
B
oAd5/3-TBD-GFP
C
Relative virus infectivity
oAd5/3-GFP
oAd5/3-TBD-GFP
p = 0.13
Dose (μM)
0 1 2 5 10 20 50 100
0 0.2 0.4 0.6 0.8 1 1.2

18

Figure S2. Evaluation of anti-adenoviral drug responsiveness of oAd5/3-TBD-GFP in comparison to the control vector using cidofovir.

To assess the safety profile of the newly developed oAd5/3-TBD-GFP vector, its responsiveness to the anti-adenoviral drug cidofovir was evaluated. HEK293 cells were treated with 5 multiplicity of infection (MOI) of either oAd5/3-GFP or oAd5/3-TBD-GFP, with or without cidofovir (0-100 μM). At 48 h post-infection, GFP expression was assessed. GFP expression decreased in a dose-dependent manner, with no significant differences between the two vectors. (**A**) Representative fluorescence and merged fluorescence images showing GFP expression in HEK293 cells treated with oAd5/3-GFP and varying concentrations of cidofovir. (**B**) Representative fluorescence and merged fluorescence images for oAd5/3-TBD-GFP under similar conditions. (**C**) Quantification of GFP expression based on data from panels (A) and (B).

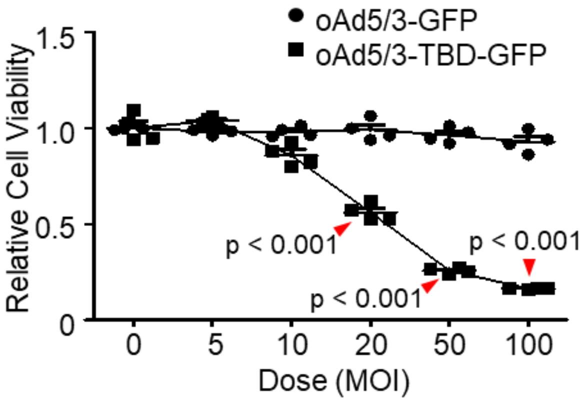

Figure S3. Assessment of antibody evasion by oAd5/3-TBD-GFP using mouse serum.

The ability of oAd5/3-GFP and oAd5/3-TBD-GFP to evade antibody-mediated neutralization was evaluated using cell viability assays in RPMI medium containing 1% mouse serum and 100 ng/mL anti-adenovirus neutralizing antibodies. Statistical comparisons were performed using a two-tailed t-test.

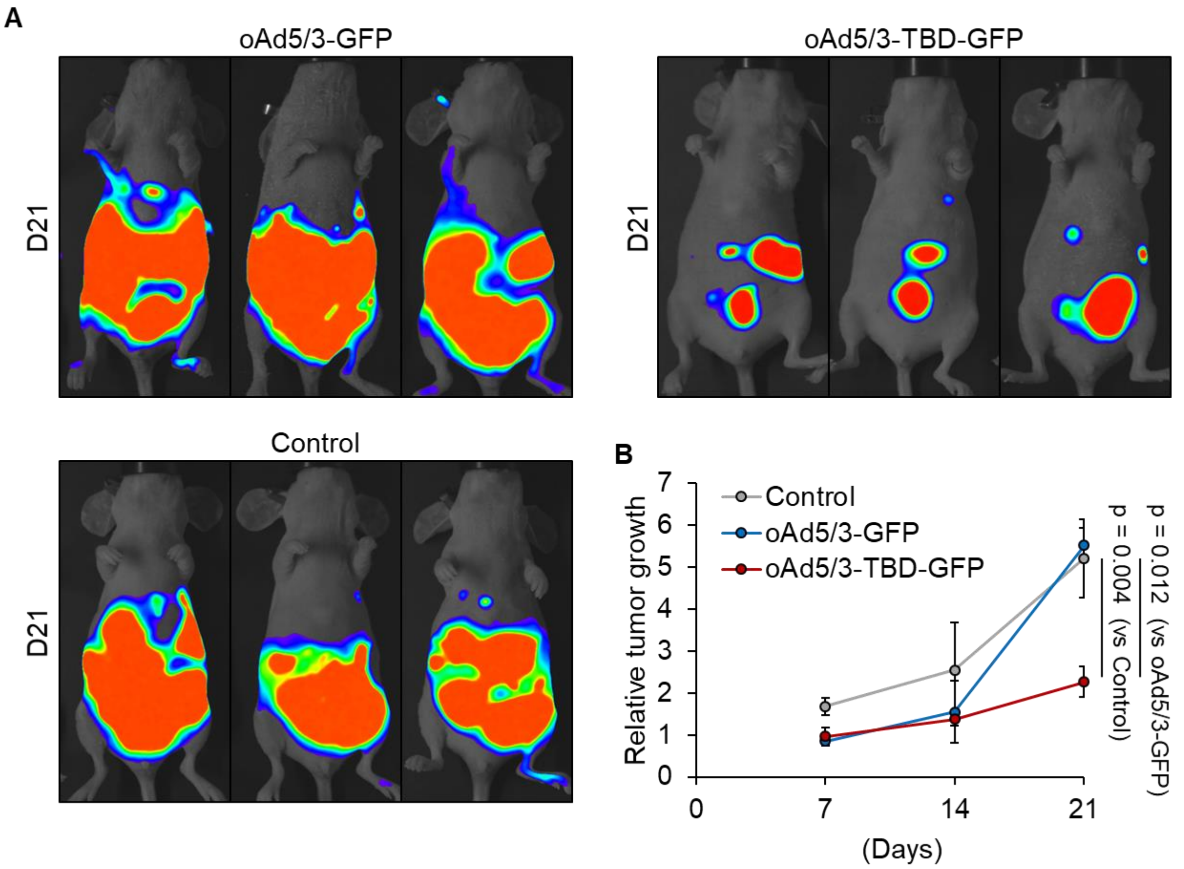

Figure S4. Efficacy of oAd5/3-TBD-GFP in a metastatic ovarian cancer mouse model.

The therapeutic efficacy of oAd5/3-TBD-GFP was evaluated in a metastatic ovarian cancer model established via intraperitoneal injection of 1×10^5 HeyA8-luc cells on day 1. Mice were intravenously injected with anti-adenovirus antibody and 5×10^8 IFU of either oAd5/3-GFP or oAd5/3-TBD-GFP on day 4. Tumor progression was monitored weekly using bioluminescent imaging with the VISQUE system. (**A**) Bioluminescent imaging of HeyA8-luc tumors at day 21 post-cell injection. (**B**) Quantification of tumor growth based on VISQUE imaging and chemiluminescence analysis.

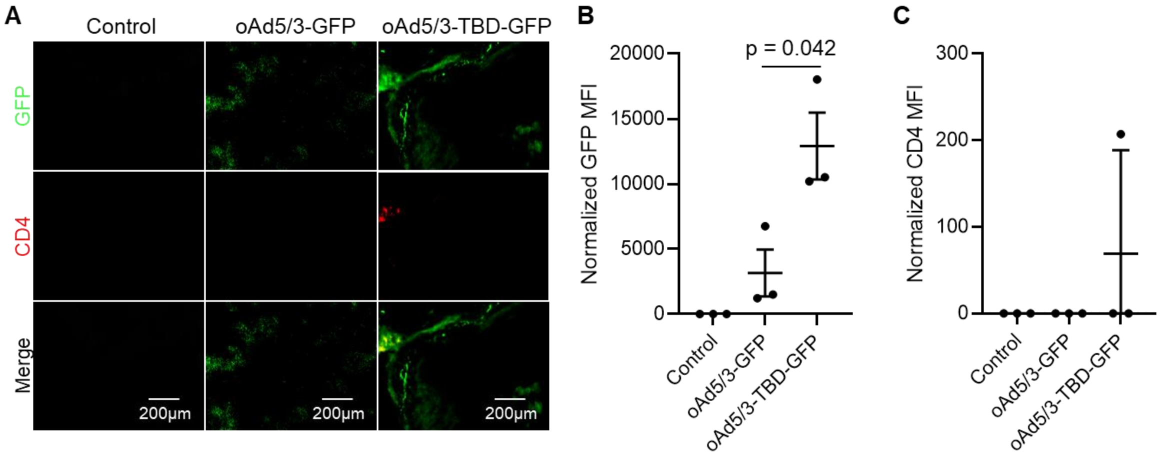

Figure S5. Analysis of CD4$^+$ T cell infiltration in tumors from Figure 8.

(**A**) Representative fluorescence images showing GFP expression (green) and CD4$^+$ T cell infiltration (red) in tumor sections stained with CD4 antibodies. (**B**) Quantification of GFP expression from panel (A). (**C**) Quantification of CD4$^+$ T cell infiltration from panel (A).
